# Dysregulation of MicroRNA-181a-5p Targets TNFAIP3 to Promote MIF-CXCR4 Signaling and Immune Inflammatory Remodeling in Chronic Myeloid Leukemia

**DOI:** 10.12688/f1000research.172236.3

**Published:** 2026-03-26

**Authors:** Noha Mohammed Saleh, Rawaa AlChalabi, Yasir Issa, Shahad Nassurat

**Affiliations:** 1 Pathology, Al Iraqia University College of Medicine, baghdad, 3, 10079, Iraq; 2 Department of Molecular and Medical Biotechnology, College of Biotechnology, Al-Nahrain University, baghdad, 3, 10079, Iraq; 3 College of Health and Medical Techniques/ Baghdad, Middle Technical University, baghdad, 3, 10079, Iraq; 4 Biology, Middle East University, baghdad, 3, 10079, Iraq

**Keywords:** Chronic myeloid leukemia, CXCR4, Immune biomarkers, Inflammation, MIF, miR-181a, NF-κB signaling, TNFAIP3.

## Abstract

**Background:**

The non-coding RNAs, particularly microRNA-181a-5p target the expression of tumor necrosis factor alpha-induced protein 3 (TNFAIP3) a key negative regulator of NF-κB signaling and affect the levels of macrophage migration inhibitory factor (MIF) and chemokine receptor type 4 (CXCR4) in chronic myeloid leukamia (CML).

**Methods:**

The study included 57 individuals with chronic myeloid leukemia (CML) and 33 healthy individuals. Hematological parameters (Hb, RBCs, WBCs and platelets) were assessed. The serum levels of MIF, TNFAIP3 and CXCR4 were measured using ELISA Technique. Quantitative real-time polymerase chain reaction was performed to assess miR-181a expression. The potential targets and immune associated pathways of miR-181a were predicted using bioinformatics tools including TargetScan, miRTarBase, STRING, DAVID, and Enrichr. Statistical analysis included ROC curve evaluation, Pearson correlation, and t-tests.

**Results:**

Compared to controls, CML patients exhibited reduced platelets, Hb and RBC while elevated WBCs recorded. There were significantly elevated serum levels of MIF and CXCR4, and reduced levels of TNFAIP3 (p<0.01) in CML patients compared to control. Moreover, higher miR-181a expression (2.28 fold, p=0.0001) recorded in CML compared to control. Positive correlations were observed between miR-181a expression and both MIF and CXCR4 levels while TNFAIP3 exhibited a reverse correlation. ROC analysis showed that MIF (AUC = 0.873) and CXCR4 (AUC = 0.929) exhibited strong diagnostic performance while TNFAIP3 (AUC = 0.142) and miR-181a-5p (AUC = 0.201) demonstrated weak accuracy consistent with their opposite expression patterns between CML patients and healthy controls.

**Conclusion:**

The findings of this study suggested that increased miR-181a expression may be associated with reduced TNFAIP3 levels and modified NF-κB related inflammatory signaling in CML. These findings support the hypothesis in which miR-181a, MIF, and CXCR4 may contribute to immune dysregulation in CML as well as diagnostic biomarkers and promising therapeutic target.

## Introduction

The Philadelphia chromosome, a key component of chronic myeloid leukemia (CML), activates signaling pathways like RAS/MAPK, PI3K/AKT, and JAK/STAT, promoting cell proliferation and suppressing apoptosis. ^1, 2^ Tyrosine kinase inhibitors (TKIs) have greatly enhanced the outlook for CML patients, leading to long-lasting remissions and a considerable increase in survival rates. ^3^ Research suggests that microRNAs (miRNAs) and other non-coding RNAs play a role in CML oncogenesis, stemness, and treatment resistance control. MicroRNAs, which are regulatory RNAs with a tiny size (about 22 nucleotides), fix to matching sequences in the 3 prim untranslated region (UTR) or coding sections of messenger RNAs (mRNAs) and suppress gene expression by mRNA degradation or translational inhibition. ^4^ MiR-181a affects cell survival and proliferation by targeting oncogenic regulators. Certain miRNA signatures have been linked to resistance to TKIs, stem cell maintenance, and leukemic transformation, and dysregulation of miRNA networks has been noted across different phases of CML progression. ^5, 6^ Due to its dual involvement in influencing apoptosis and immunological signaling, miR-181a has garnered significant attention among the miRNAs implicated in hematological malignancies. MicroRNA-181a has been reported to have regulatory behavior in hematological malignancies with variable expression patterns observed across disease phases, treatment status, and cellular compartments. Leukemic cell survival, immune evasion, and inflammatory responses are all regulated by the NF-κB signaling system. ^7^ In CML, the promotion of an inflammatory milieu and the survival of leukemic stem cells are caused by abnormal NF-κB activation contributes to disease progression and persistence. ^8, 9^ An essential negative regulator of the NF-κB pathway is TNFAIP3 (tumor necrosis factor alpha induced protein 3), or A20 which limits inflammation and apoptosis resistance. ^10^ The production of inflammatory mediators can be increased when TNFAIP3 expression is either lost or suppressed, as this can augment NF-κB activity. The NF-κB pathway includes MIF (macrophage migration inhibitory factor) and CXCR4 (C-X-C chemokine receptor type 4), two downstream targets that may promote the migration of leukemic cells, the remodeling of the microenvironment, and resistance to treatment. ^11^ While CXCR4 facilitates the chemotactic migration of leukemic cells to protect bone marrow niches, MIF is a pro-inflammatory cytokine that promotes tumor growth and immune evasion. In hematologic malignancies, upregulation of MIF and CXCR4 has been linked to a worse prognosis and treatment failure. ^12^ Few studies have explored the role of miR-181a and other non-coding RNAs in the context of CML research in Iraq. ^13^ Due to the genetic diversity in Iraq and the increasing number of hematologic malignancies including CML further local research into miRNA-mediated regulation mechanisms is required.

### Aims of the study

The objective of this research was to compare the miR-181a expression levels and blood concentrations of MIF, TNFAIP3, and CXCR4 in CML patients with those in healthy controls. The study also aimed to use bioinformatics analysis to determine the downstream impacts on pathways mediated by NF-κB and the regulatory link between miR-181a and TNFAIP3.

## Materials and methods

### Study design

This case-control experimental study was designed to compare the gene expression of miR-181a and the serum concentrations of selected biomarkers (MIF, TNFAIP3, CXCR4) between CML patients and healthy controls. Bioinformatics analysis was additionally performed to investigate molecular interactions between miR-181a and its target genes.

### Ethical approval

The study protocol was approved by the Iraqi Ministry of Health/Medical City/Center of Hematology, Baghdad, Iraq (approval number 41008, dated 14-11-2024). Informed written consent was obtained from all participants. The study was carried out from November 2024 to November 2025.

### Sample size and subjects

The study included 90 people: 33 healthy controls who were the same age and sex as the participants and 57 patients with chronic-phase CML. Hematology clinics and hospitals in Iraq’s capital city of Baghdad were the sites of participant recruitment. As no a priori sample size determination could be conducted prior to participant recruitment, a posterior sample size estimation was done using G*Power version 3.1. Given a total sample size of 90 participants (33 in group 1, 57 in group 2) with an alpha level of 0.05 and a statistical power of 0.90, the posterior analysis suggested that the study was able to detect an effect size of d = 0.72, which is considered a medium-to-large effect based on Cohen’s criteria.

### Inclusion and exclusion criteria

Participants were to be adults aged >20 years who had recently been diagnosed with chronic-phase CML according to molecular, hematological, and clinical criteria. None of the health controls had a family history of autoimmune diseases, chronic inflammatory illnesses, or hematologic malignancies were included in this study. Patients with CML in its rapid or blast crisis phases, individuals using immunosuppressive medication, or those suffering from autoimmune or infectious disorders at the same time were excluded in this study.

### Hematological parameters

Using the ADVIA 2120i Hematology Analyzer (Siemens Healthcare Diagnostics, Germany) at the Research Unit, College of Health and Medical Techniques/Baghdad, Middle Technical University, we immediately after blood collection measured complete blood counts (CBC), which include Hb, RBCs, WBCs, and PLTs.

### Serum biomarker measurement

Enzyme-linked immunosorbent assay (ELISA) kits (Sunglong Biotech Co., China) were used to quantify the serum concentrations of TNFAIP3, MIF, and CXCR4.

### RNA extraction

Following the manufacturer’s instructions, 500 μL of serum samples were treated with TRIzolTM Reagent (Thermo Scientific, USA) to extract total RNA, which includes short RNAs. The RNA pellet was rinsed with 70% ethanol and resuspended in nuclease-free water after being separated with chloroform and isopropanol for RNA precipitation. In order to determine the amount and quality of the RNA, a Quantus Fluorometer (Promega, USA) was used.

### cDNA synthesis

Complementary DNA (cDNA) was produced from RNA extraction by means of the GoScriptTM Reverse Transcription System (Promega, USA). Reverse transcription with random primers and the GoScript enzyme was carried out at 42°C for 60 minutes after the RNA and primer were denaturated at 70°C for 5 minutes. The enzyme was then inactivated at 70°C for 15 minutes to complete the operation.

### Primers used in this study

The sequences for miR-181a and RNU43 were obtained from the iRbase database ( https://www.mirbase.org/) and primers were designed consequently. The following primers were manufactured by Macrogen (Korea) and used in the study: miR-181a Forward Primer (miR-181a-F2): 5′-TGTTTGACCATCGACCGTTG-3′. miR-181a Reverse Transcription Primer (miR-181a-RT): 5′-GTTGGCTCTGGTGCAGGGTCCGAGGTATTCGCACCAGAGCCAACGGTACA-3′. RNU43 Reverse Transcription Primer (RNU43-RT): 5′-GTTGGCTCTGGTGCAGGGTCCGAGGTA TTCGCACCAGAGCCAACAATCAG-3′. RNU43 Forward Primer (RNU43-F): 5′-GTGAACTTATTGACGGGCG-3′. Universal Reverse Primer: 5′-GTGCAGGGTCCGAGGT-3′. The optimal annealing temperature was 55°C for each set of primers. To make working solutions, the concentration of primers was 10 pmol/μL, and the stock concentration was 100 pmol/μL, both of which were adjusted in nuclease free water.

### Gene expression quantification (Real-Time PCR)

The relative expression of miR-181a was measured via qRT-PCR on a Mic qPCR Cycler (Bio Molecular Systems, Australia) with the GoTaq qPCR Master Mix (Promega, USA). A cDNA template, specific primers, and SYBR Green master mix were all components of each 20 μL PCR reaction. Initial denaturation was done at 95°C for 5 minutes, followed by 40-cycles of 95°C for 20 seconds, 55°C for 20 seconds (fluorescence collection), and 72°C for 20 seconds as the thermal cycling conditions. The relative expression was determined using the 2^-ΔΔCt method, also known as the Livac method, after normalizing the expression levels against the house keeping gene RNU43.

### Bioinformatics analysis

Bioinformatics was used to predict the miR-181a-TNFAIP3 interaction utilizing the miRTarBase and TargetScan databases. Utilizing the STRING database, protein-protein interaction networks incorporating TNFAIP3, MIF, and CXCR4 were generated. The DAVID and Enrichr platforms were used to perform functional enrichment analysis on biological processes and signaling pathways.

### Statistical analysis

The SPSS software (version 27) was used for all statistical analyses. The assumptions of normality of data distribution were preformed using the Shapiro–Wilk test and for the equality of variances across groups Levene’s test were used. Because these assumptions were met, an independent samples t-test was performed to assess the mean differences between the two groups. An independent samples t-test was used to examine the differences between the control group and the CML patients. The correlation analysis was used to investigate the association between miR-181a expression and serum biomarker values. To evaluate the diagnostic adequacy of miR-181a and the investigated biomarkers, ROC curve analysis was carried out. Statistical significance was established when the p-value was less than 0.05. The relative gene expression of the target genes was assessed using the Livak (2^−ΔΔCt) method. ^13^

## Results

### Demographic characteristics of the study population

There was no significant difference in the age and sex distributions between the control. Percentages were derived from group totals (CML n = 57: 28 males, 29 females; Control n = 33: 16 males, 17 females). The sex distribution therefore corresponds to 49.1% male/50.9% female in CML and 48.5% male/51.5% female in controls (pairs sum to 100%). A chi-square test for the sex and age variable showed no difference across groups in sex distribution. For age (4 categories), the omnibus test showed χ ^2^ = 0.280, df = 3, p = 0.964 (NS) and demonstrated no disparity in age distributions, as indicated in Table 1 (p > 0.05).

**Table 1.** The demographic characteristics of CML and control.

Group	Sex	CML patients	Control	Chi-square	P-value
Sex	Male	28 (49.1%)	16 (48.5%)	0.112	0.821 NS
	Female	29 (50.9%)	17 (51.5%)	0.043	0.932 NS
Age (Years)	20-30	2 (3.5%)	1 (3.0%)	0.536	0.91 NS
	30-40	11 (19.3%)	6 (18.2%)	0.633	0.892 NS
	40-50	28 (49.1%)	15 (45.5%)	1.231	0.724 NS
	>50	16 (28.1%)	11 (33.3%)	1.000	1.00 NS

NS: No significant differences (p > 0.05). Report one chi-square and p-value per variable (Sex; Age), not per category row.

### Profile of study participants

Table 2 showed that there were statistically significant changes in major hematological parameters between healthy controls and CML patients. The CML group had significantly lower hemoglobin levels (8.74 ± 0.20 g/dL) when compared to the control group (11.41 ± 0.48 g/dL; p = 0.0000). Patients had significantly reduced RBC counts (3.51 ± 0.04 ×10 ^6^/μL) compared to controls (4.69 ± 0.12 ×10 ^6^/Μl, p = 0.0000). Compared to the controls, the CML group had significantly higher WBCs counts (27.87 ± 0.77 ×10 ^3^/μL) with a p-value of 0.0000. In comparison to the controls, patients had significantly decreased platelet counts (174.69 ± 2.49 ×10 ^3^/μL) with a p-value of 0.0001. Chronic myeloid leukemia is characterized by severe hematologic dysregulation, as shown in Table 2.

**Table 2.** Hematological profile in CML compared to control.

Parameter	CML patients (Mean ± SE)	Controls (Mean ± SE)	P-value
Hb	8.74 ± 0.20	11.41 ± 0.48	0.0000 **
RBC	3.51 ± 0.04	4.69 ± 0.12	0.0000 **
WBC	27.87 ± 0.77	14.96 ± 1.76	0.0000 **
Platelets	174.69 ± 2.49	226.25 ± 10.97	0.0001 **

** Significant differences, p < 0.01.

### Biomarker expression

**Figure 1.**
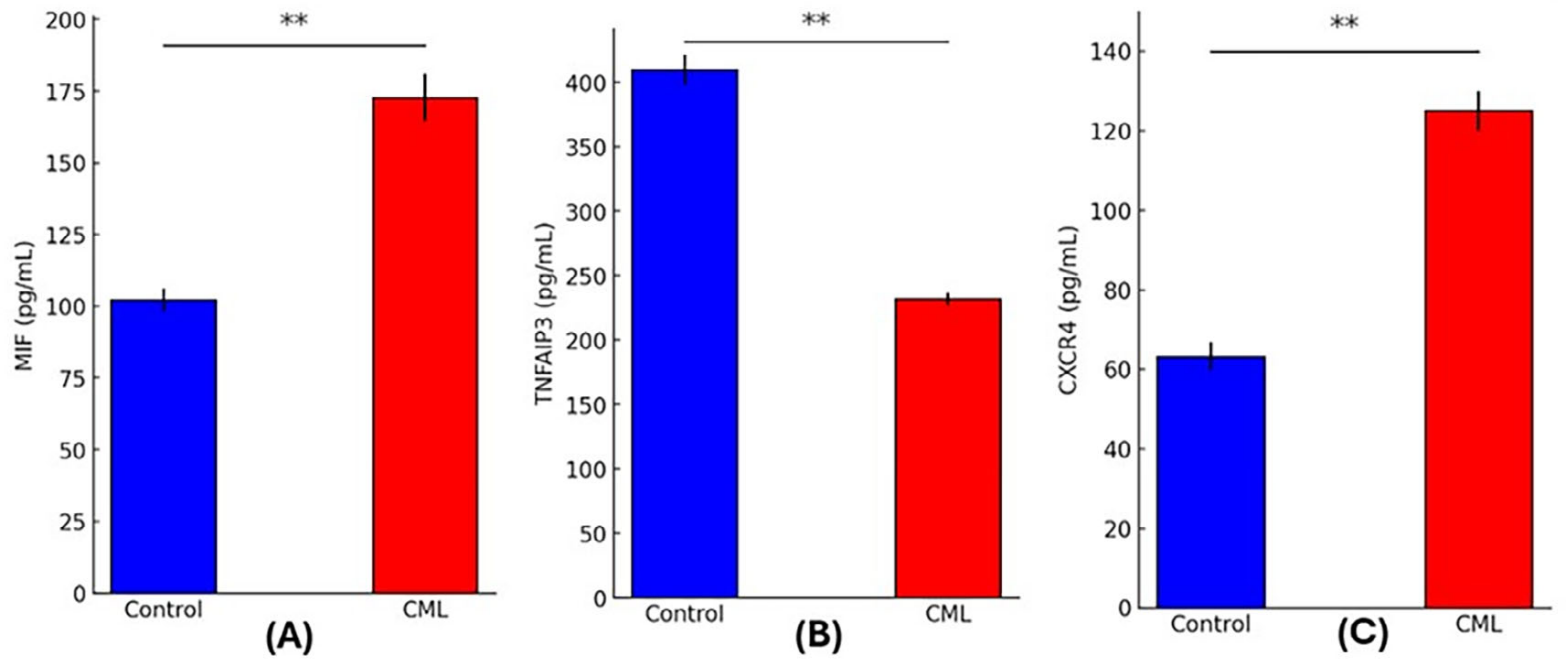
Serum levels of inflammatory and signaling biomarkers in CML patients and healthy controls. (A) MIF, (B) TNFAIP3, and (C) CXCR4 in CML patients (red bars) versus control subjects (blue bars). (p < 0.01, **).

Comparing CML patients with healthy controls using quantitative analysis of circulating biomarkers showed striking differences. Signifying increased macrophage activation, MIF levels were significantly higher in the CML group (172.73 ± 8.22 pg/mL) in comparison to the controls (102.25 ± 3.88 pg/mL; p = 0.0000). The TNFAIP3 levels were considerably lower in CML patients (231.91 ± 4.85 pg/mL) compared to controls (409.89 ± 11.54 pg/mL; p = 0.0000), which may indicate that the anti-inflammatory regulation through the NF-κB pathway was suppressed. Patients had significantly increased CXCR4 expression (125.06 ± 5.05 pg/mL) compared to controls (63.30 ± 3.49 pg/mL; p = 0.0000) suggesting that there was an increase in chemokine receptor signaling and potential leukemic cell movement. The inflammatory and immunological dysregulation linked to CML pathogenesis is highlighted by these biomarker profiles, as shown in Figure 1.

### Relative expression of miR-181 in CML patients and control

Patients with chronic myeloid leukemia (CML) had significantly higher transcript levels of miR-181 (2.28) than healthy controls fold change (1) according to relative quantification analysis (p = 0.0001) reported in Table 3.

**Table 3.** Gene expression of miR-181 in CML patients and controls.

Group	RNU43 CT (Mean ± SD)	miR-181 CT (Mean ± SD)	ΔCT (Mean ± SD)	ΔΔCT (Mean ± SD)	2^-ΔΔCT (Mean ± SD)	Fold change
Control	15.83 ± 2.10	15.21 ± 1.78	-0.62 ± 1.35	0.00 ± 0.00	1.00 ± 0.00	1
CML	14.91 ± 1.98	13.10 ± 1.55	-1.81 ± 1.22	-1.19 ± 0.45	2.28 ± 0.51	2.28
P-value	0.0912 NS	0.061 NS	0.023 *	0.0001 **	0.0001 **	–

NS: Not significant.

* p < 0.05.

** p < 0.01.

### Correlation between miR-181 expression and biomarkers

In the study population, Pearson correlation analysis demonstrated strong positive correlations between miR-181 fold-change and serum levels of MIF (r = 0.617, p < 0.0001) and CXCR4 (r = 0.630, p < 0.0001). The expression of miR-181 was shown to be strongly correlated negatively with TNFAIP3 levels (r = -0.758, p < 0.0001). Based on these results it appears that CML-specific inflammatory and chemokine signaling patterns are linked to miR-181 expression. As reported in Table 4.

**Table 4.** Correlation between miR-181-fold change and serum biomarker levels.

Biomarker	Correlation with miR-181 (r)	P-value
MIF	0.617	0.0000 **
TNFAIP3	-0.758	0.0000 **
CXCR4	0.630	0.0000 **

** p < 0.001.

### Diagnostic performance of biomarkers and miR-181

**Figure 2.**
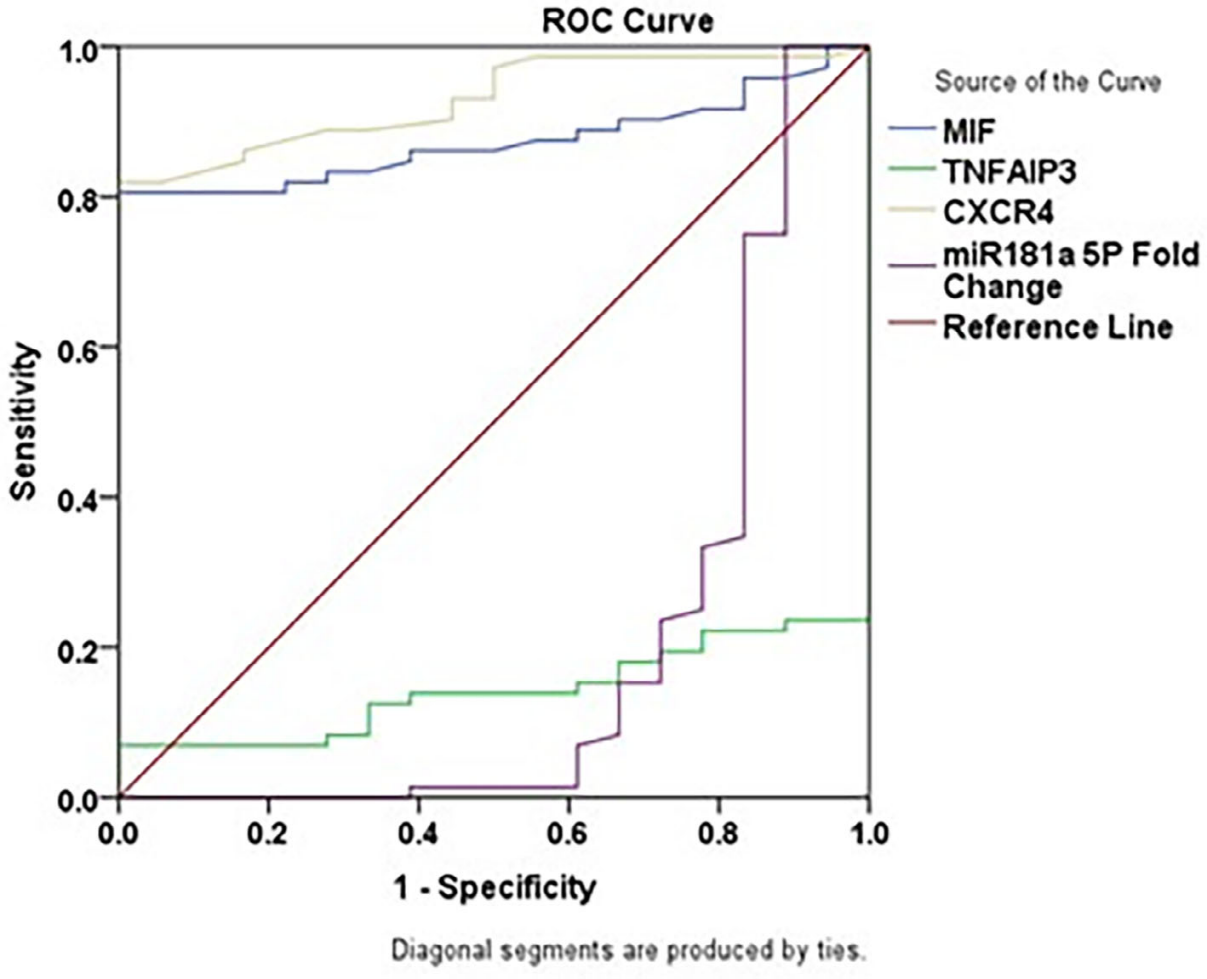
ROC curves of MIF, TNFAIP3, CXCR4, and miR-181a-5p in CML patients.

In CML patients, ROC analysis showed that the sensitivity, specificity, AUC, cutoff, and p-value for MIF were 80.6%, 100.0%, 0.873, 80.10 pg/mL and p<0.001, respectively; for CXCR4 were 81.9%, 100.0%, 0.929, 68.10 pg/mL, and p<0.001, respectively; for TNFAIP3 were 6.9%, 100.0%, 0.142, 523.32 pg/mL, and p<0.001, respectively; and for miR-181a-5p were 100%, 11.1%, 0.201, 1.00 and p<0.001, respectively. These results are presented in Table 5 and Figure 2.

**Table 5.** Diagnostic performance of biomarkers and miR-181.

Group	Sensitivity %	Specificity %	95% Confidence Interval	Area ± SE	Cutoff	P value
MIF	80.6	100.0	0.803 – 0.944	0.873 ± 0.036	80.10	0.000 *
TNFAIP3	6.9	100.0	0.067 – 0.217	0.142 ± 0.038	523.32	0.000 *
CXCR4	81.9	100.0	0.878 – 0.980	0.929 ± 0.026	68.10	0.000 *
miR-181a-5p	100.0	11.1	0.044 – 0.358	0.201 ± 0.080	1	0.000 *

* p < 0.001

### miR-181 and biomarker interactions

**Bioinformatics analysis of miR-181a target interactions and regulatory pathways**

**Figure 3.**
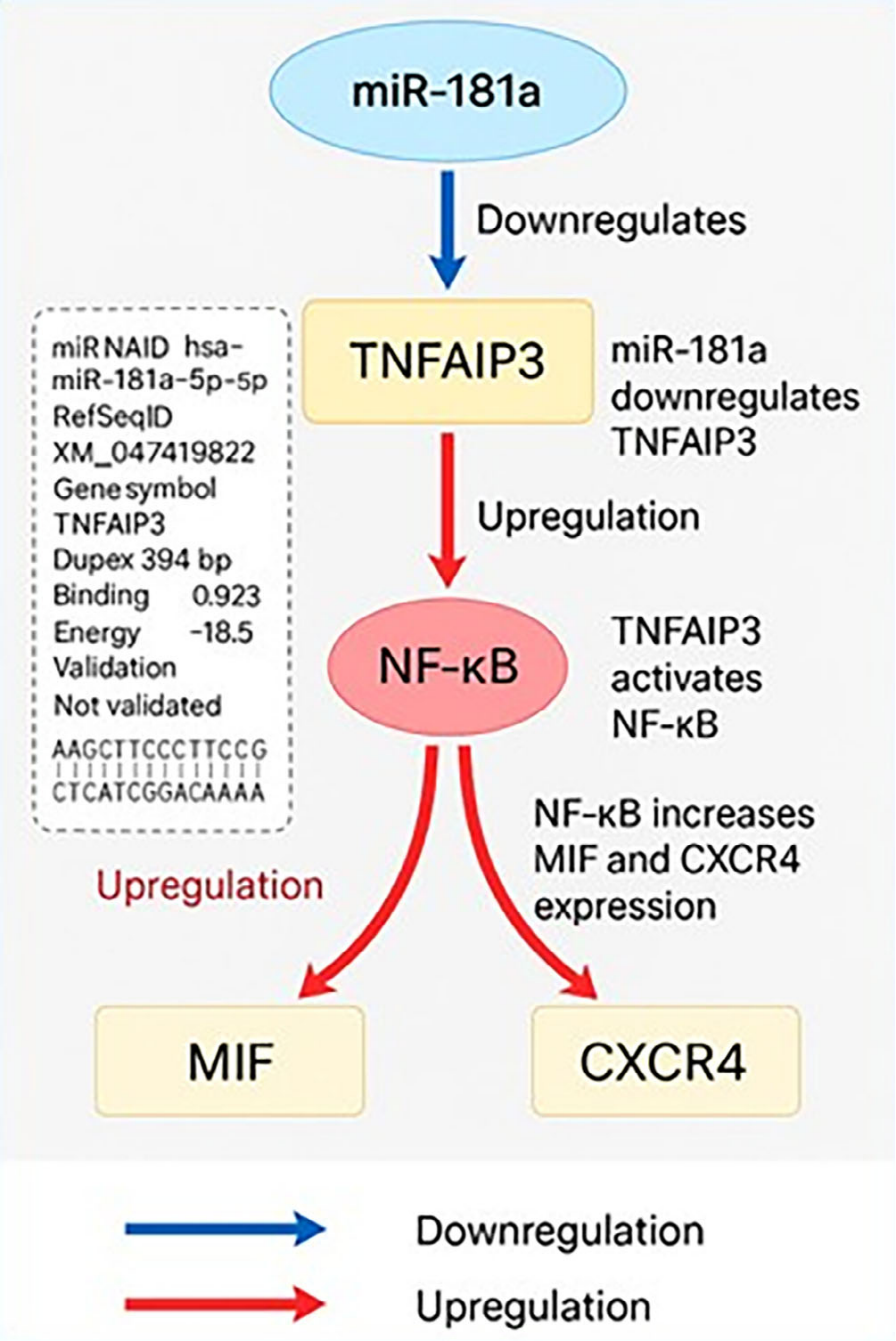
Bioinformatic model of miR-181a-mediated post-transcriptional regulation of TNFAIP3 and its downstream impact on NF-κB signaling, MIF, and CXCR4 expression in CML.

The TNFAIP3 gene was predicted to be a likely direct target of hsa-miR-181a-5p in silico ( Figure 3). Target prediction was carried out with miRWalk 3.0 (version 2018 release), TargetScanHuman 8.0, and miRDB (version 6.0). The prediction was made with a minimum free energy (MFE) threshold ≤ -15 kcal/mol, binding probability ≥ 0.70, and seed region complementarity of ≥ 7 consecutive nucleotides. In miRWalk, miR-181a-5p had a noteworthy binding site within the CDS of TNFAIP3 mRNA at positions 394–406 with an MFE of -18.5 kcal/mol and a binding probability of 0.923. The predicted duplex has 11 consecutive base pairs in a 12-base region indicating a stable and efficient interaction. However, TNFAIP3 was not predicted to be a target by TargetScan or miRDB which underscores the differences in the databases. Functionally TNFAIP3 is a known negative regulator of NF-κB signaling. Suppression of TNFAIP3 by miR-181a-5p would relieve this inhibition and thus, promote NF-κB activity. Functional enrichment and network analyses were performed by STRING (version 12.0) and NDExBio which showed MIF and CXCR4 to be downstream targets of NF-κB. There were no sites of direct binding predicted for miR-181a-5p with MIF or CXCR4 in the prediction databases, but the results outlined above indicate that miR-181a-5p is also increased MIF and CXCR4 levels by indirectly targeting and downregulating TNFAIP3 thus stimulating inflammatory signaling pathways involved in the pathogenesis of CML.

## Discussion

Chronic myeloid leukemia (CML) is characterized by a disturbance of normal hematopoiesis, which is supported by the hematological findings noted in the study which include anemia, leukocytosis, and thrombocytopenia. ^14^ These changes, which show that BCR-ABL1 oncogenic signaling is the root cause of bone marrow failure, are similar to what has been shown in other studies conducted in Iraq and in large-scale cohorts around the world. More evidence is pointing to the important function of microRNAs (miRNAs) in controlling cancer pathways, inflammatory reactions, and treatment resistance in CML. ^15– 17^ Rather than indicating a direct oncogenic function the elevated expression of miR-181a observed in CML patients suggests a potential regulatory involvement in immune and inflammatory signaling pathways associated with the disease. The observed elevation in newly diagnosed, untreated CML cases is consistent with findings that demonstrate context-dependent dynamic regulation of miR-181a, ^18^ even though some studies, including reports from worldwide datasets, have shown a downregulation of miR-181a in TKI-sensitive patients. ^19^ Bioinformatics analyses predicted TNFAIP3 as a putative target of miR-181a suggesting that increased miR-181a expression may contribute to reduced TNFAIP3 levels and subsequent modulation of NF-κB signaling however this relationship requires functional experimental validation. Previous functional investigations have shown that leukemic cell survival and inflammatory signaling are both enhanced when TNFAIP3 activity is lost, and this molecular relationship is in agreement with those findings. ^20^ This regulation model was further supported by correlation analysis which showed that miR-181a expression was positively connected with MIF and CXCR4 levels and inversely correlated with TNFAIP3. ^21^ Prior research has shown that MIF enhances leukemic cell survival and accelerates chemokine-mediated migration via CXCR4 which is supported by the increased levels of MIF seen in CML patients. Simultaneously, the study’s identification of CXCR4 overexpression lends credence to the crucial function of the CXCR4/SDF-1 axis in preserving leukemic stem cells within protected bone marrow niches, which in turn contributes to the perpetuation of disease and resistance to therapy. ^22, 23^ These results are consistent with what researchers in Iraq and around the world have found, which further proves that these pathways are important in the development of CML everywhere. ^24^ The results are new and significant for the area research environment because few studies have thoroughly assessed the diagnostic performance of miR-181a in conjunction with inflammatory biomarkers in CML. This study fills a gap in the literature by offering a thorough integrated examination of the relationship between miRNA dysregulation and inflammatory signaling pathways in CML patients, in contrast to earlier sparse studies from Iraq. ^25– 27^ Based on the results of this study, a possible mechanism is that an increase in miR-181a inhibits TNFAIP3, which in turn activates NF-κB signaling and increases the levels of inflammatory and chemotactic effectors including MIF and CXCR4. Further research with bigger patient cohorts and functional validation assays is needed to confirm these findings and investigate their therapeutic implications of the miR-181a/TNFAIP3/MIF/CXCR4 axis in CML pathogenesis. Novel approaches to treating treatment resistance and enhancing clinical outcomes in CML patients may be revealed by future studies that focus on this regulatory network.

In conclusion, this study demonstrates altered expressions of miR-181a and dysregulated serum levels of TNFAIP3, MIF, and CXCR4 in patients with chronic myeloid leukemia. These findings suggest a potential association between miR-181a expression and inflammatory signaling pathways relevant to CML.

## Data Availability

Zenodo: Dysregulation of MicroRNA-181a-5p Targets TNFAIP3 to Promote MIF-CXCR4 Signaling and Immune Inflammatory Remodeling in Chronic Myeloid Leukemia. https://doi.org/10.5281/zenodo.17653441. ^28^ This project contains the following underlying data:
•
**CML.xlsx:** This single dataset contains all raw and processed data used in the statistical analyses, including hematological parameters (Hb, RBC, WBC, PLT), serum biomarker measurements (MIF, TNFAIP3, CXCR4), and qPCR data (RNU43 Ct, miR-181a Ct, ΔCt, ΔΔCt, and fold-change values). •
**Participant questionnaire:** Used during sample collection. Data are available under the terms of the Creative Commons Attribution 4.0 International License (CC-BY 4.0).
