# Supplementary material for: Dysregulation of MicroRNA-181a-5p Targets TNFAIP3 to Promote MIF-CXCR4 Signaling and Immune Inflammatory Remodeling in Chronic Myeloid Leukemia
Source: F1000Res. 2026 Mar 26;14:1460. Originally published 2025 Dec 26. [Version 3] doi: 10.12688/f1000research.172236.3 (PMC13054252; doi:10.12688/f1000research.172236.3)
Supplement: Peer review history

# Reviewer report: Reviewer response for version 3

**DOI:** 10.5256/f1000research.197132.r471436

**Published:** 2026-04-06

**Authors:** Dan Jones, Ryan Stevens

**Affiliations:** 1 Ohio State University James Comprehensive Cancer Center, Columbus, Ohio, USA

Saleh et al present a case control study comprising 57 CML patients (vs 33 age/sex-matched controls) assessing miR-181a expression in serum by qRT-PCR as compared to serum levels of a NF-kB growth factor inhibitor (TNFAIP3) and a chemokine and a chemokine receptor (MIF and CXCR4, respectively) by ELISA. They show that miR-181a transcripts are upregulated in the serum of stable phase untreated CML as were MIF and CXCR4 protein levels whereas TNFAIP3 was decreased (previously shown in one prior study cited). Using bioinformatic analysis of the TNFAIP3 transcript, the Authors postulate that MiR-181a may be directly downregulating TNFAIP3 leading to activation of NF-kB pathway and chemokine upregulation.

 Major Points to Address:

- Abstract - This sentence is confusing and should be rewritten since TNFAIP3 and miR-181a just don’t predict for CML in your study: “ROC analysis showed that MIF (AUC =0.873) and CXCR4 (AUC = 0.929) exhibited strong diagnostic performance while TNFAIP3 (AUC = 0.142) and miR-181a-5p (AUC = 0.201) demonstrated weak accuracy consistent with their opposite expression patterns between CML patients and healthy controls.”   You could just mention MIF and CXCR4 and leave out the other two in the abstract and just discuss their performance in the results.
- Methods: Was RNA really extracted from “serum” (not buffy coat or plasma) using Trizol?  If so, I highly doubt the levels of miRs are going to be reflective of the cellular component.  If this is true, this needs to be prominently mentioned in results such as “significantly higher transcript levels of miR-181 (2.28) **in serum**, and also mentioned this in the abstract. The discussion should note that serum miRs may not reflect cellular levels, as a possible problem with the study
- Discussion: There is too direct a connection made between MIF and CXCR4 levels and NF-kB activation due to TNFAIP3 decreases. Both of these chemokine pathways are also highly regulated by other growth factors.  Please add some caveats about this connection to the Discussion.  You should also mention that miR-181 is known to regulate other important growth regulatory pathways in CML cells which could also be contributing to these observations.
- Also, CML is characterized by increased IMMATURE myeloid cells compared to normal hematopoiesis. Since immature myeloid cells have markedly increased CXCR4 and MIF (compared to neutrophils), the correlations observed may be reflective of the disease and not caused by miR-181 directly. Add something about this to the Discussion,
- Given the lack of functional validation of the in silico analysis of TNFAIP3, I would also add to the Discussion, literature on the connection between miR-181a in leukemic cell models (there is data in CLL and other leukemic models).
- Sample size is small so your comment in the Discussion on needing validation in a larger cohort should also be in the abstract.
- There are still spelling grammar, spacing, and wording issues throughout.
  - For example in Introduction: “bind to” not “fix to” matching sequences; “3’ prime” not “3 prim”; “contributing” not “contributes” to disease progression and persistence.
  - Write “miR-181a” consistently throughout as far as capitalization if at sentence start.
  - Please grammar and spell check the entire document.

Minor Points to Address:

- Title: I would say “MIF and CXR4 signaling (not “MIF-CXCR4”) since these are not in the same chemokine family
- Methods: Mention how many (if any) of the CML patients had prior treatment and the median treatment interval before the sample used (even short treatments can greatly affect NF-kB and chemokine pathways).
- Methods: Mention whether replicate measurement were made by qRT-PCR and/or by ELISA.
- Results:  I would remove “Signifying increased macrophage activation,” since MIF is expressed widely (DCs, CML myeloid cells) since its elevation isn’t proof of macrophage activation.
- Discussion: Add paragraph breaks, to make the comments easier to read (as well as adding discussion of the points above).

Is the work clearly and accurately presented and does it cite the current literature?

Partly

If applicable, is the statistical analysis and its interpretation appropriate?

Partly

Are all the source data underlying the results available to ensure full reproducibility?

Yes

Is the study design appropriate and is the work technically sound?

Yes

Are the conclusions drawn adequately supported by the results?

Partly

Are sufficient details of methods and analysis provided to allow replication by others?

Yes

Reviewer Expertise:

CML, immunology, molecular diagnostics (Jones), molecular diagnostics (Ryan Stevens, who contributed to this review).

We confirm that we have read this submission and believe that we have an appropriate level of expertise to confirm that it is of an acceptable scientific standard, however we have significant reservations, as outlined above.

# Reviewer report: Reviewer response for version 2

**DOI:** 10.5256/f1000research.195767.r455867

**Published:** 2026-02-24

**Authors:** Ikhwan Rinaldi

**Affiliations:** 1 Cipto Mangunkusumo Hospital, Central Jakarta, Universitas Indonesia, Jakarta, Indonesia

Thank you for the revisions. However, the following key concerns remain unaddressed and should be resolved:

 1. The revised manuscript still does not provide a justification for the chosen sample size, nor does it report any a priori power or sample size calculations. Please clarify whether a power analysis was performed and, if not, provide a rationale for the selected sample size and discuss its implications for the statistical robustness of the findings.

 2. Independent t-tests continue to be used as the primary statistical method. However, the manuscript does not report whether assumptions of normality and homogeneity of variance were assessed. Please specify how these assumptions were evaluated or justify the continued use of parametric tests.

Is the work clearly and accurately presented and does it cite the current literature?

Yes

If applicable, is the statistical analysis and its interpretation appropriate?

Yes

Are all the source data underlying the results available to ensure full reproducibility?

Yes

Is the study design appropriate and is the work technically sound?

Yes

Are the conclusions drawn adequately supported by the results?

Yes

Are sufficient details of methods and analysis provided to allow replication by others?

Partly

Reviewer Expertise:

Hemato-oncology

I confirm that I have read this submission and believe that I have an appropriate level of expertise to confirm that it is of an acceptable scientific standard, however I have significant reservations, as outlined above.

# Response

**Published:** 2026-02-24

**Authors:** Yasir Issa

**Affiliations:** Reasarch Unit, Middle Technical University, Baghdad, Karrada street 13, Iraq

In this revised version, we have further strengthened the statistical methodology in response to reviewer comments. Specifically, we have clarified the assessment of assumptions underlying parametric testing by explicitly reporting the use of the Shapiro–Wilk test and Q–Q plots to evaluate normality, as well as Levene’s test to assess homogeneity of variances. We have also indicated that Welch’s correction was applied when appropriate.

 In addition, a post-hoc power analysis has been included to justify the adequacy of the sample size. The calculated statistical power (1−β = 0.98) confirms that the study was sufficiently powered to detect significant group differences. We have also clarified the presentation of descriptive statistics, specifying that data are expressed as mean ± standard error (SE) to reflect the precision of the estimated mean. These additions enhance the transparency, robustness, and methodological clarity of the study without altering the original results or conclusions.

# Response

**Published:** 2026-03-12

**Authors:** Yasir Issa

**Affiliations:** Reasarch Unit, Middle Technical University, Baghdad, Karrada street 13, Iraq

thank you for your valuable comments. We have now clarified the statistical considerations in the revised manuscript.

 A sensitivity analysis was conducted using G*Power (v3.1) based on the available sample size (33 participants in group 1 and 57 in group 2). With an alpha level of 0.05 and statistical power of 0.90, the analysis indicated that the study was capable of detecting an effect size of d = 0.72, which corresponds to a medium-to-large effect according to Cohen’s criteria. This information has now been added to the Statistical Analysis section.

 In addition, we have clarified that the assumptions required for parametric testing were evaluated prior to analysis. Normality of the data was assessed using the Shapiro–Wilk test, and homogeneity of variance between groups was examined using Levene’s test. Independent-samples t-tests were applied after confirming that these assumptions were satisfied.

# Reviewer report: Reviewer response for version 2

**DOI:** 10.5256/f1000research.195767.r455868

**Published:** 2026-02-23

**Authors:** haijiao Zhang

**Affiliations:** 1 Oregon Health & Science University, Portland, Oregon, USA

The revision adequately addressed the previous concerns.

Is the work clearly and accurately presented and does it cite the current literature?

Partly

If applicable, is the statistical analysis and its interpretation appropriate?

Partly

Are all the source data underlying the results available to ensure full reproducibility?

Partly

Is the study design appropriate and is the work technically sound?

Partly

Are the conclusions drawn adequately supported by the results?

Partly

Are sufficient details of methods and analysis provided to allow replication by others?

Partly

Reviewer Expertise:

NA

I confirm that I have read this submission and believe that I have an appropriate level of expertise to confirm that it is of an acceptable scientific standard.

# Reviewer report: Reviewer response for version 1

**DOI:** 10.5256/f1000research.189951.r448884

**Published:** 2026-01-23

**Authors:** haijiao Zhang, Anna He

**Affiliations:** 1 Oregon Health & Science University, Portland, Oregon, USA; 2 Division of Oncological Sciences, Oregon Health & Science University Knight Cancer Institute (Ringgold ID: 608031), Portland, Oregon, USA

**Dysregulation of MicroRNA-181a-5p Targets TNFAIP3 to Promote MIF-CXCR4 Signaling and Immune Inflammatory Remodeling in Chronic Myeloid Leukemia**

 Noha Mohammed Saleh ^1^, Rawaa AlChalabi ^2^, Yasir Issa ^3^, Shahad Nassurat ^4^

**Comments to the Author:**

 The study by Noha Mohammed Saleh et al. evaluated gene expression of miR-181a and serum concentrations of biomarkers MIF, TNFAIP3, and CXCR4 in CML patients compared to healthy controls, assessing interactions between miR-181a and target genes. They demonstrated that miR-181a expression was significantly upregulated in CML patients and directly targets TNFAIP3. A proposed mechanism is that increased miR-181a expression inhibits TNFAIP3, activating NF-κB signaling and regulating downstream inflammatory mediators such as MIF and CXCR4. They conclude that miR-181a plays a crucial role in CML pathogenesis through regulation of TNFAIP3 and other downstream inflammatory mediators.

 The following major and minor questions should be addressed or corrected accordingly.

**Major Questions**

 This study is primarily descriptive and does not include experimental validation. Accordingly, the conclusions should be interpreted as hypothesis-generating, with proposed mechanisms, biomarkers, and downstream targets considered putative and requiring further molecular and functional validation.

 Conclusion such as mentioned in the discussion that: A possible oncogenic role was highlighted by the study finding that miR-181a expression was significantly upregulated in CML patients compared to healthy controls.

 This claim is inaccurate; oncogenic properties were not assessed, and no relationship between oncogenic activity and miR-181a expression was demonstrated.

 In addition, at the end of the discussion, the author wrote that: In conclusion, the study revealed that miR-181a plays a crucial role in CML pathogenesis via regulating TNFAIP3 and other downstream inflammatory mediators. These results expand the study molecular knowledge of CML and point to potential areas for future diagnostic and treatment advancements, especially for patients in Iraq and the surrounding area.

 This statement is inaccurate, as the study is descriptive and reports only increased miR-181a levels in CML patients, along with the potential diagnostic value of MIF and CXCR4 in CML; therefore, concluding that miR-181a plays a crucial role in CML pathogenesis is an overstatement.

**Minor Criteria**

 1) Some parts of the writing need careful editing, such as punctuation and spacing in the Introduction and Discussion. Additionally, in the Introduction, abnormal NF-κB activation “improves the disease duration” of CML; however, the positive connotation of “improves” should be noted. The footnote to Table 1 seems to have additional unnecessary text. In the Abstract Conclusion, “MIFpresent3” is incorrect. Collectively, this study suggests a novel pathogenic mechanism involving immune dysregulation in CML through identification of miR-181a, MIF, and CXCR4 in immune modulation and inflammation.

 2) There is an inconsistency between the Introduction, References, and Results. In the Introduction, the authors state that “CML stem/progenitor cells downregulate miR-181a expression, making them more susceptible to apoptosis and TKIs,” but no citation is provided to support this claim. Moreover, this statement is inconsistent with the study’s findings showing that miR-181a is upregulated in CML patients in your cohort.  

 3) In the paragraph below, the reported correlation coefficients (R = 0.617 and R = 0.630, both p < 0.05) indicate strong correlations rather than a “slight association.”

 Correlation between miR-181 expression and biomarkers

 In the study population, the Pearson correlation analysis showed a slight association between the miR-181-fold change and the levels of MIF (r = 0.617, p < 0.0001) and CXCR4 (r = 0.630, p < 0.0001). The expression of miR-181 was shown to be strongly correlated negatively with TNFAIP3 levels (r = -0.758, p < 0.0001). Based on these results it appears that CML-specific inflammatory and chemokine signaling patterns are linked to miR-181 expression. As reported in Table 4.

Is the work clearly and accurately presented and does it cite the current literature?

Partly

If applicable, is the statistical analysis and its interpretation appropriate?

Partly

Are all the source data underlying the results available to ensure full reproducibility?

Partly

Is the study design appropriate and is the work technically sound?

Partly

Are the conclusions drawn adequately supported by the results?

Partly

Are sufficient details of methods and analysis provided to allow replication by others?

Partly

Reviewer Expertise:

Leukemia, translation oncology, drug resistance mechanisms.

We confirm that we have read this submission and believe that we have an appropriate level of expertise to confirm that it is of an acceptable scientific standard, however we have significant reservations, as outlined above.

# Reviewer report: Reviewer response for version 1

**DOI:** 10.5256/f1000research.189951.r446680

**Published:** 2026-01-08

**Authors:** Ikhwan Rinaldi

**Affiliations:** 1 Cipto Mangunkusumo Hospital, Central Jakarta, Universitas Indonesia, Jakarta, Indonesia

The manuscript addresses the role of miR-181a-5p–mediated regulation of TNFAIP3 and its downstream inflammatory signaling pathways in chronic myeloid leukemia (CML). Several issues should be addressed before the manuscript can be considered for indexing.

 Abstract

 In the Methods section, please clearly state the study design (case–control), the main laboratory techniques employed (qRT-PCR and ELISA), and the statistical tests used.

 In the Results section, if possible, key quantitative findings should be summarized more explicitly, including effect sizes or fold changes with corresponding p-values for miR-181a, MIF, TNFAIP3, and CXCR4.

 Methods and Results

 The sample size justification is not described. Please clarify whether a priori sample size or power calculations were performed to ensure adequate statistical power.

 The statistical analysis relies heavily on independent t-tests. Please confirm that assumptions of normality and variance homogeneity were assessed, or alternatively justify the use of non-parametric tests where appropriate.

 Figure 2 (ROC curves) would benefit from clearer annotation. Please consider adding arrows or labels indicating the AUC values for each biomarker directly on the figure.

 Abbreviations should be consistently defined in all tables and figure captions.

 Discussion

 The authors are encouraged to expand the limitations section to include the single-center design, relatively modest sample size, and population specificity, which may affect generalizability.

Is the work clearly and accurately presented and does it cite the current literature?

Yes

If applicable, is the statistical analysis and its interpretation appropriate?

Yes

Are all the source data underlying the results available to ensure full reproducibility?

Yes

Is the study design appropriate and is the work technically sound?

Yes

Are the conclusions drawn adequately supported by the results?

Yes

Are sufficient details of methods and analysis provided to allow replication by others?

Partly

Reviewer Expertise:

Hemato-oncology

I confirm that I have read this submission and believe that I have an appropriate level of expertise to confirm that it is of an acceptable scientific standard, however I have significant reservations, as outlined above.
